# Podocyte protease activated receptor 1 stimulation in mice produces focal segmental glomerulosclerosis mirroring human disease signaling events

**DOI:** 10.1016/j.kint.2023.02.031

**Published:** 2023-03-20

**Authors:** Carl J. May, Musleeha Chesor, Sarah E. Hunter, Bryony Hayes, Rachel Barr, Tim Roberts, Fern A. Barrington, Louise Farmer, Lan Ni, Maisie Jackson, Heidi Snethen, Nadia Tavakolidakhrabadi, Max Goldstone, Rodney Gilbert, Matt Beesley, Rachel Lennon, Rebecca Foster, Richard Coward, Gavin I. Welsh, Moin A. Saleem

**Affiliations:** 1 Bristol Renal, University of Bristol, Bristol, UK; 2 Renal Medicine and Nephrology, Southampton General Hospital, University Hospital Southampton, Southampton, UK; 3 Pathology Department, Gloucestershire Royal Hospital, Gloucester, UK; 4 Wellcome Trust Centre for Cell Matrix Research, Division of Cell Matrix Biology and Regenerative Medicine, Faculty of Biology, Medical and Health Sciences, School of Biological Sciences, University of Manchester, Manchester, UK; 5 Department of Paediatric Nephrology, Bristol Royal Hospital for Children, Bristol, UK

**Keywords:** circulating factor, nephrotic syndrome, PAR-1, podocyte, proteases

## Abstract

About 30% of patients who have a kidney transplant with underlying nephrotic syndrome (NS) experience rapid relapse of disease in their new graft. This is speculated to be due to a host-derived circulating factor acting on podocytes, the target cells in the kidney, leading to focal segmental glomerulosclerosis (FSGS). Our previous work suggests that podocyte membrane protease receptor 1 (PAR-1) is activated by a circulating factor in relapsing FSGS. Here, the role of PAR-1 was studied in human podocytes *in vitro*, and using a mouse model with developmental or inducible expression of podocyte-specific constitutively active PAR-1, and using biopsies from patients with nephrotic syndrome. *In vitro* podocyte PAR-1 activation caused a pro-migratory phenotype with phosphorylation of the kinase JNK, VASP protein and docking protein Paxillin. This signaling was mirrored in podocytes exposed to patient relapse-derived NS plasma and in patient disease biopsies. Both developmental and inducible activation of transgenic PAR-1 (NPHS2 Cre PAR-1^Active+/−^) caused early severe nephrotic syndrome, FSGS, kidney failure and, in the developmental model, premature death. We found that the non-selective cation channel protein TRPC6 could be a key modulator of PAR-1 signaling and TRPC6 knockout in our mouse model significantly improved proteinuria and extended lifespan. Thus, our work implicates podocyte PAR-1 activation as a key initiator of human NS circulating factor and that the PAR-1 signaling effects were partly modulated through TRPC6.

Idiopathic nephrotic syndrome (INS) is one of the most difficult clinical conditions to manage in nephrology. Breakdown of the glomerular filtration barrier results in massive proteinuria, with the underlying trigger not well understood. A proportion of patients will not respond to any therapy, leading inevitably to renal failure and transplantation. In patients without a detectable genetic mutation, up to 50% suffer recurrence of disease.^1^ This leads to the reduced lifespan of the graft, and often after graft loss, the patient is deemed untransplantable given the very high risk of further recurrences.

There is ample evidence that recurrent INS is caused by an as yet unknown circulating factor, likely produced by the immune system. Despite decades of study, the identity of such circulating factor(s) remains unknown.^1^ Here we have tested the hypothesis that the circulating factor works through podocyte protease-activated receptor 1 (PAR-1).

PARs 1 to 4 are part of the well-known family of G protein–coupled receptors and are activated by serine proteases. PAR-1 and PAR-2 have been demonstrated to be expressed in the glomerulus, and immunohistochemical staining shows high PAR-1 expression in the podocytes.^2^ Circulating plasma carries many proteases with diverse biological roles, which are tightly regulated by cognate antiproteases.

We previously showed data suggesting that there is excess circulating protease activity in the plasma of post-transplant INS patients with active post-transplant disease (referred to as relapse plasma) that can signal to podocytes resulting in vasodilator-stimulated phosphoprotein (VASP) phosphorylation and increased podocyte motility, with the signaling being podocyte specific.^3^ This effect is not present in response to remission plasma from the same patients or control plasma. Small, interfering RNA knockdown of PARs in podocytes demonstrated a role for the PAR-1 protease receptor in this response to relapse plasma.

We hypothesized that there is a protease(s) that is present in the circulation that becomes dysregulated early in the pathogenesis of INS (either by upregulation or loss of a cognate antiprotease). Both T cells and memory B cells have been postulated as sources of the circulating factor,^4,5^ and our subsequent work suggests that the T helper cell 17 (Th17) subset could be a source of circulating proteases.^6^ This increased activity of the circulating protease stimulates PAR-1 and induces specific signaling cascades.

To test this at the receptor level, we now activated PAR-1 *in vitro* in conditionally immortalized human podocytes. We then used this signaling data to assess pathologic signaling in human nephrotic patient kidney biopsies and NPHS2 Cre PAR-1^Active+/−^ mice.

Furthermore, we hypothesized that podocyte TRPC6 (transient receptor potential cation channel subfamily c member 6) mediates PAR-1 signaling and is activated in response to human plasma or PAR-1 agonism. TRPC6 is an ion channel that binds to the podocyte-specific membrane protein podocin,^7^ and activating mutations in this channel in patients are able to cause focal segmental glomerular sclerosis (FSGS).^8^ Both gain-of-function and loss-of-function mutations are known to cause FSGS.^9^ In addition, it has been shown in podocytes that thrombin treatment can increase intracellular calcium concentrations. This increase is blocked by treatment with a TRPC3/6 inhibitor.^10^

## Methods

### PAR-1 active construct

The PAR-1 active construct was a kind gift of Dr. Shaun Coughlin, University of California San Francisco.^11^ Briefly, all serine and threonine residues in the C-terminal tails have been substituted for alanines. This renders the PAR-1 receptor phosphor-null at the C-terminal tail. This form of the receptor is defective in both shutoff and agonist-triggered internalization.

### Animals

All animal experiments and procedures were approved by the UK Home Office in accordance with the Animals (Scientific Procedures) Act 1986, and the *Guide for the Care and Use of Laboratory Animals* was followed during experiments. SV129 transgenic mice were generated by Genoway. These mice were bred with NPHS2 Cre hemizygous mice or NPHS2 rtTA Tet O Cre mice to generate developmental and inducible animals, respectively.

The Pod Cre PAR-1 mice were crossed with whole-body TRPC6 knockout (KO) mice on a C57/Bl6 background. These mice were back-crossed to enrich for SV129.

Inducible transgenic mice were fed 2 mg/ml doxycycline in 5% sucrose in their drinking water to activate the inducible transgene. Treatment started between 4 and 6 weeks of age and continued for 3 weeks. The start of doxycycline treatment has been designated week 0. The animals were culled at 3, 10, and 12 weeks, and their kidneys were examined.

There is sufficient detail included in this paper to comply with the ARRIVE (Animal Research: Reporting of In Vivo Experiments) guidelines for the reporting of animal data.

### Cell culture

Human wild-type (WT) podocytes were conditionally immortalized using the SV40T antigen system. This allows podocytes to proliferate at the permissive temperature of 33 °C and differentiate at 37 °C.^12^ Podocytes were cultured as previously described.^13^ Mouse podocyte cell lines were generated from a TRPC6 KO mouse on a C57 Bl/6 background as per the protocol detailed in a previous publication.^13^ Human glomerular endothelial cells were generated in-house and were conditionally immortalized using the same SV40T system as used in the podocytes. Mouse cell lines were generated by isolating glomeruli *ex vivo*, as described previously.^14^

### Treatments

Cultured podocytes were treated with PAR-1 agonist (Peptides International PAR-3665-PI) at a concentration of 15 μM for the indicated times. Podocytes were also treated with thrombin as a PAR-1 agonist (Sigma; #T6884) at a dose of 15 units/μl again for the indicated times. Podocytes were also treated with nephrotic patient plasma as previously described.^3^ Each plasma pair was obtained from the same patient.

### Western blotting

Total protein lysates were extracted using a sodium dodecylsulfate lysis buffer supplemented with phosphatase inhibitor cocktails 2 and 3 (P5726 and P0044; Sigma Aldrich).

Blots were probed with primary antibodies: phospho–c-Jun N-terminal kinase (pJNK) Thr183/Tyr185 (#9251; Cell Signaling Technology), *P* p38 mitogen-activated protein kinase (MAPK) Thr180/Tyr182 (#9211; Cell Signaling Technology), β-actin (#A4700; Sigma), Phospho Paxillin Ser 178 (ab 193677; Abcam). All primaries were used following a 1:1000 dilution in 3% bovine serum albumin and incubated with the blot at 4 °C overnight.

Primary antibodies were probed using a horseradish peroxidase–conjugated species-specific secondary antibody: either rabbit (#A0545; Sigma) or mouse (#A9044; Sigma). Secondaries were used following a 1:10,000 dilution in 3% bovine serum albumin and incubated with the blot for 1 hour at room temperature.

The membranes were incubated with a synthetic analog of peroxide and with a signal enhancer, which are mixed in a 1:1 ratio (Geneflow; K1-0070).

Blots were imaged using the Amersham Imager 600 from GE Healthcare Life Sciences.

### Densitometry

The densitometry in this study has been calculated using ImageJ. All densitometry represents the optical density of the band. This value is normalized to both a counterpart-actin load control and the mean of the untreated control bands of the antibody in question.

### Scratch assay

Podocytes were cultured in 6-well plates. Scratch assays were performed once the podocytes had spent 14 days at the nonpermissive temperature of 37 °C. In brief, the media were aspirated, and a mechanical wound was inflicted by scratching the monolayer with a pipette tip. Podocytes were then washed in phosphate-buffered saline twice to remove any debris and promigratory factors. The wells were then treated, and the scratch area was imaged after 0 (control) and 12 hours using a Leica DMIRB microscope and a Zeiss Axiocam Erc 5S camera. The area of the clear zone was measured over time, and podocyte migration was assessed by reduction in the area, indicating more motile cells.

### Mice

PAR-1^Active+/−^ mice were generated by Genoway, France. The NPHS2 Cre and NPHS2 Cre rtTA mice were a gift from Professor Susan Quaggin. TRPC6 KO mice were purchased from the Jackson Laboratory.

### Tissue processing and staining

Kidneys were harvested and immersed in formalin before being paraffin embedded. Kidneys were sectioned by our in-house histology facility. Masson’s trichrome stain was performed using a kit (HT15-1KT; Sigma), and periodic acid–Schiff stain was also performed using a kit (395B; Sigma).

### Immunohistochemistry quantification

The first 6 to 8 glomeruli alighted upon during looking at the slide were imaged. The glomeruli were manually isolated from the rest of the image. The immunohistochemistry (IHC) was quantified using an open-source plugin for ImageJ called the “IHC profiler.” Therefore, the IHC profiler measures the glomerular signal only. The plugin is described in detail here.^15^

### Pathology screening

A pathologist (MB) was sent 48 images of the glomeruli from the developmental mice, 24 from each of the control and PAR-1^Active+/−^ mice. MB was blinded to the genotype associated with each image. Images were scored for glomerulosclerosis.

### Calcium influx assay

A more detailed description of this assay can be found here.^16^ Briefly, the R_norm_ was plotted against time, and the peak to baseline ratios of R_norm_ were calculated as a measure of the maximal effect on [Ca^2+^]_i_.

### Statistical analysis

Graphs and statistical analyses were compiled and performed using GraphPad Prism (GraphPad Software). The following asterisks have been used to denote statistical significance: ^⋆^*P* ≤ 0.05, ^⋆⋆^*P* ≤ 0.01, ^⋆⋆⋆^*P* ≤ 0.001, ^⋆⋆⋆⋆^*P* ≤ 0.0001.

### Human biopsies

Biopsy tissue was obtained via the UK Nephrotic Syndrome Study, NephroS, housed within the UK Renal Rare Disease Registry, RaDaR.

## Results

### PAR-1 is highly expressed in the podocyte and its *in vitro* activation is detrimental

**Figure 1:**
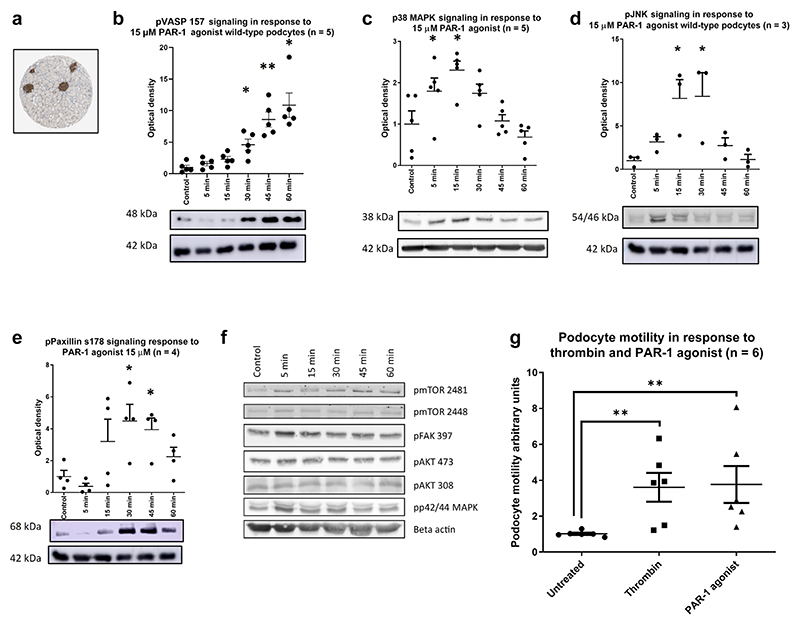
Protease-activatedreceptor 1 (PAR-1) is highly expressed in the podocyte, and its activation is detrimental. (**a**) Data from Sigma’s Protein Atlas show an enrichment of PAR-1 expression within the glomeruli of the kidney. All densitometry graphs show the optical density of the band, normalized to the control and corrected by β-actin load control. An example blot is shown beneath each graph. Wild-type conditionally immortalized human podocytes were treated with a PAR-1 agonist at a dose of 15 μM for the indicated time points. There was significant phosphorylation of VASP (**b**), p38 mitogen-activated protein kinase (MAPK) (**c**), JNK (**d**), and Paxillin (**e**) Bonferroni’s multiple comparison test, *P ≤ 0.05, **P ≤ 0.01, ***P ≤ 0.001, ****P ≤ 0.0001. A range of pathways were interrogated that were not significantly stimulated (**f**). A wound-healing assay was performed to assess the ability of the PAR-1 agonist to induce a motile phenotype (**g**). Data shown n = 3 in duplicate, normalized to untreated control. Both the PAR-1 agonist and thrombin treatments significantly increase podocyte motility (1-tailed Mann-Whitney test *P =* 0.0011 and 0.0022, respectively). pJNK, phospho–c-Jun N-terminal kinase; pVASP, phospho–vasodilator-stimulated phosphoprotein.

The PAR-1 receptor is highly expressed by the podocyte, as shown in the Sigma protein atlas^17^ (Figure 1a), strikingly in Nephrocell RNA expression data^18^ and in single-cell RNA databases.^19^

Conditionally immortalized human podocytes were treated with PAR-1 agonist (Figure 1b–e). This agonist is a short peptide and is highly specific for the PAR-1 receptor. This treatment stimulated a significant increase in the phosphorylation of VASP at serine 157 (Figure 1b). Previous work published by the group has shown a similar response to NS patient plasma, which was reduced after knockdown of the PAR-1 receptor.^3^ In addition, we now show that p38 MAPK, JNK, and Paxillin all displayed significant increases in phosphorylation in response to PAR-1 agonist treatment (Figure 1c–e, respectively). The phospho-VASP (pVASP), JNK, and Paxillin phosphorylation events are podocyte specific; no such phosphorylation was seen in PAR-1 agonist–treated glomerular endothelial cells (Supplementary Figure S1). However, phosphorylation of p38 MAPK was observed showing that the glomerular endothelial cells are PAR-1 sensitive.

Phosphorylation of paxillin at serine 178 is associated with an increase in cell motility.^20^ Several other signaling pathways were interrogated but revealed no significant stimulation in response to PAR-1 agonist (Figure 1f). Wound-healing assays revealed that PAR-1 agonist treatment significantly increased podocyte motility (Figure 1g), which is considered a surrogate of increased podocyte effacement *in vivo*.^21^

### PAR-1–associated signaling pathways are activated in response to nephrotic plasma and are modulated via TRPC6

**Figure 2:**
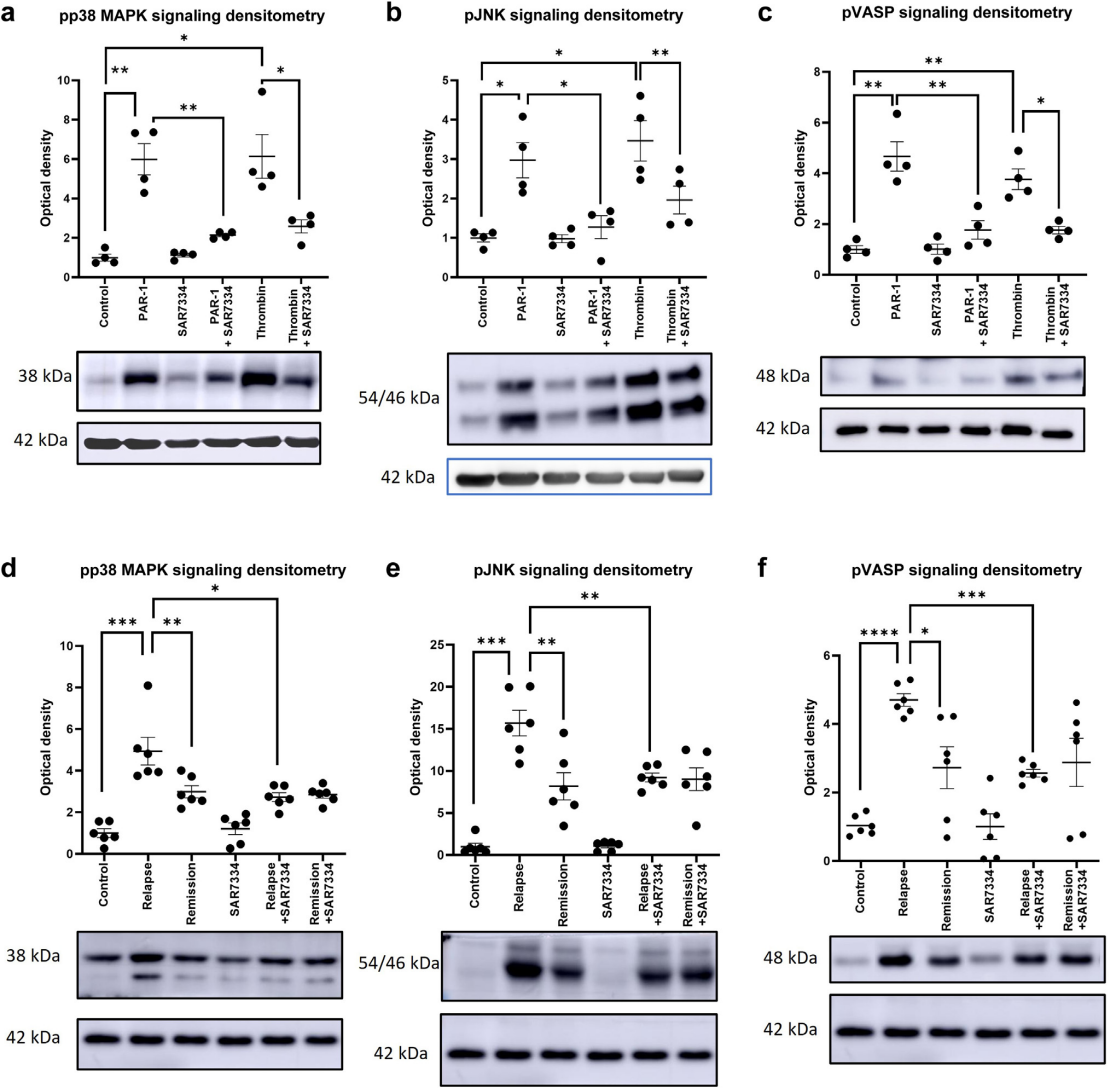
Protease-activated receptor 1 (PAR-1)–associated signaling pathways are activated in human nephrotic syndrome. PAR-1 agonist treatment at a concentration of 15 μM showed significant stimulation of (**a**) p38 mitogen-activated protein kinase (MAPK), (**b**) JNK, and (**c**) vasodilator-stimulated phosphoprotein (VASP) signaling pathways (Bonferroni’s multiple comparison test). The transient receptor potential cation channel subfamily c member 6 (TRPC6) inhibitor SAR7334 (10 nM with 30-minute preincubation) was capable of significantly dampening the response of the podocyte to PAR-1 agonist treatment (**a–c**) (Bonferroni’s multiple comparison test). Podocytes treated with relapse plasma demonstrated significant stimulation of (**d**) pp38 MAPK, (e) phospho–c-Jun N-terminal kinase (pJNK), and (**f**) phospho–vasodilator-stimulated phosphoprotein (pVASP) (Bonferroni’s multiple comparison test) compared with podocytes treated with remission plasma. A further treatment with SAR7334 significantly decreased the stimulation (**d–f**) (paired 1-way *t* test). The densitometry shown in (**a**)–(**c**) is based on 4 western bots, whereas that shown in (**d**)–(**f**) is based on 6, normalized to β-actin load control and relative to control lane. A representative blot is shown beneath each graph. *P ≤ 0.05, **P ≤ 0.01, ***P ≤ 0.001, and ****P ≤ 0.0001.

PAR-1 activating peptide treatments were repeated along with a TRPC6 inhibitor (SAR 7334 Tocris).^22^ Stimulation with PAR-1 agonist significantly stimulated the p38 MAPK (Figure 2a), JNK (Figure 2b), and VASP (Figure 2c) pathways. The inhibitor significantly reduced the signaling response to PAR-1 agonist treatment for p38 MAPK, JNK, and VASP. Thrombin (27 μM) significantly stimulated the p38 MAPK, JNK, and VASP signaling pathways (Each Bonferroni’s Multiple Comparison Test). SAR7334 (10 nM with 30-minute preincubation) treatment significantly reduced p38 MAPK, JNK, and VASP stimulation.

Patient relapse plasma also significantly stimulated the p38 MAPK, JNK, and VASP pathways compared with remission plasma treatment (Figure 2d–f). This signaling response could be significantly reduced using the TRPC6 inhibitor SAR7334.

### PAR-1–associated signaling pathways are activated in human nephrotic syndrome

**Figure 3:**
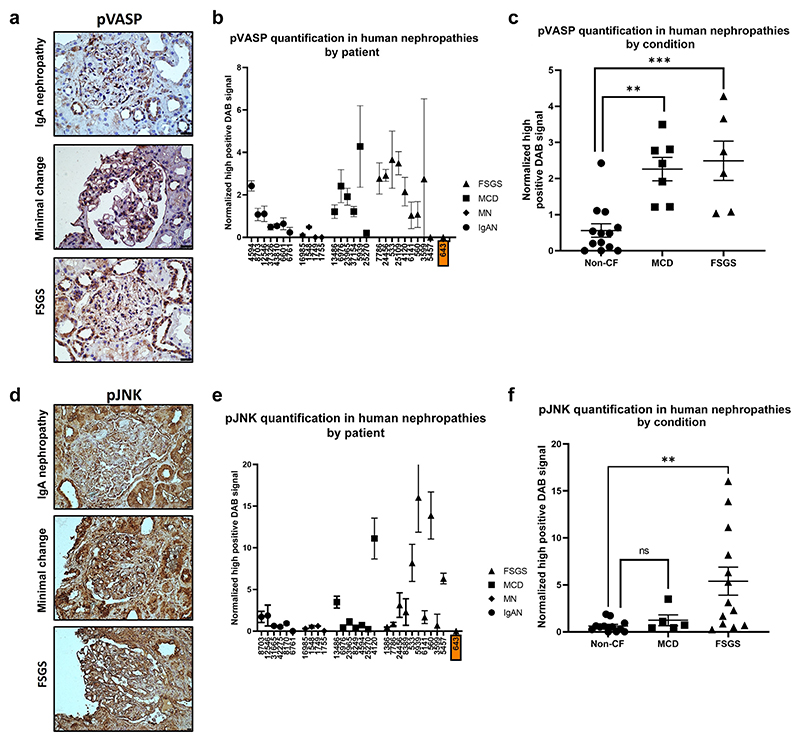
Protease-activated receptor 1 (PAR-1)–associated signaling pathways in human kidney biopsies. Human biopsy samples were stained for (**a**) phospho–vasodilator-stimulated phosphoprotein (pVASP) and (**d**) phospho–c-Jun N-terminal kinase (pJNK). The tissue was sourced from patients diagnosed with either IgA nephropathy (IgAN), membranous nephropathy (MN), minimal change disease (MCD), or focal segmental glomerular sclerosis (FSGS). Subpanels (**b**) and (**e**) show all patients tested and their replicates with the error bars showing the SEM for signal within each individual patient. Subpanels (**c**) and (**f**) show the means for each patient by disease type; here the error bars show the SEM within each disease type. There is a possible role for a circulating factor in MCD and FSGS. Although there was evident renal damage in IgAN and MN, this damage is probably not caused by the activity of the postulated circulating factor. (**c**) pVASP was significantly higher in patients with MCD and FSGS relative to patients with non–cystic fibrosis (CF) (patients with IgAN and MN) (*P =* 0.001 Bonferroni’s multiple comparison test). (**f**) There was also significantly more glomerular pJNK in FSGS glomeruli relative to non-CF (*P =* 0.021 Bonferroni’s multiple comparison test). (**b,e**) Patient identifier shown in an orange box has FSGS with a known genetic cause. *P ≤ 0.05, **P ≤ 0.01, ***P ≤ 0.001, and ****P ≤ 0.0001. DAB, 3,3′-diaminobenzidine. To optimize viewing of this image, please see the online version of this article at www.kidney-international.org.

Biopsies from patients with NS were obtained via the UK Renal Rare Disease Registry, RaDaR (Supplementary Figure S2). The biopsies were stained for pVASP, pJNK, and pPaxillin. Significant increases in the glomerular phosphorylation of VASP were seen in patients with minimal change disease and FSGS (pretransplant) compared with those with a noncirculating factor disease, IgA nephropathy (Figure 3a–c). Patient 643 has Frasier syndrome, which is a genetic condition caused by a *WT1* mutation that causes FSGS. Interestingly, there was no pVASP signal in these biopsies. A significant increase in pJNK was seen in patients with FSGS versus patients with IgA. Again patient 643 exhibited no pJNK signal (Figure 3d–f).

### Developmental activation of podocyte PAR-1 *in vivo* is highly detrimental

Overactivation of PAR-1 *in vivo*, exclusively in the podocyte, caused glomerular damage with histologic features that closely resemble human FSGS pathogenesis.

**Figure 4:**
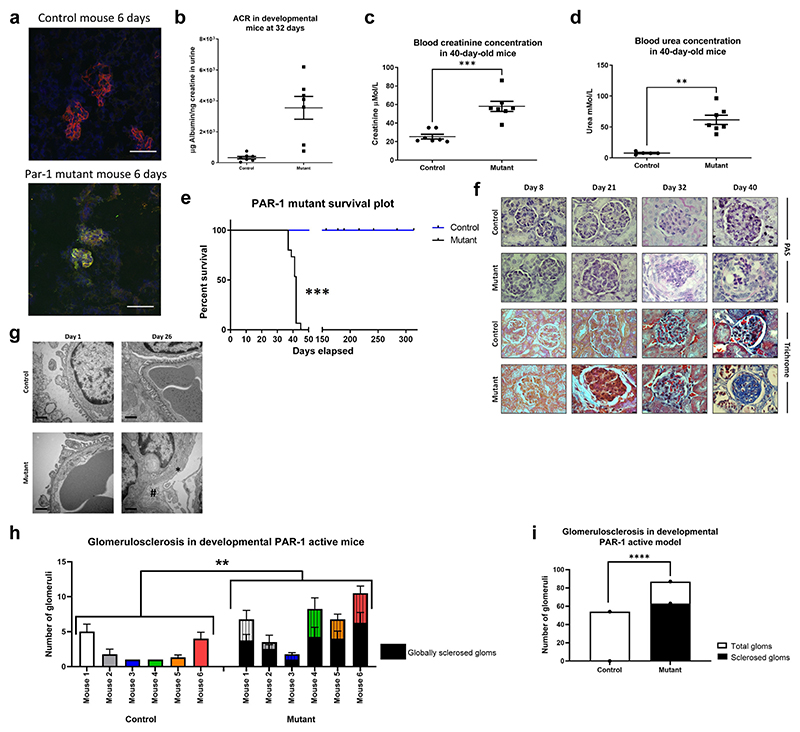
Developmental activation of podocyte protease-activated receptor 1 (PAR-1) *in vivo* is highly detrimental. (**a**) The transgenic form of PAR-1 is tagged with the synthetic peptide FLAG. This allows for the distinction between endogenous PAR-1 and the transgenic PAR-1. Kidneys from 6-day-old mice were harvested and flash frozen. The sections were stained using immunofluorescence. The blue channel shows 4′,6-diamidino-2-phenylindole, the red channel shows nephrin, and the green channel shows FLAG. There is no expression of FLAG in the control mice. The FLAG is clearly visible in the mutant mice. This shows that the transgene is being expressed. (**b**) These animals are overtly proteinuric at 32 days (*P =* 0.0005, 1-way unpaired *t* test). (**c,d**) They also have significantly higher levels of creatinine (1-tailed Mann-Whitney test, *P =* 0.0003) and urea in their blood (1-tailed Mann-Whitney test, *P =* 0.013). (e) The NPHS2 Cre PAR-1^Active+/−^ mice die around 40 days of age (controls n = 19, median survival 195.4 days, SD 49.46 days; mutants n = 15, median survival 40.8 days, SD 2.23 days; log-rank Mantel-Cox test, *P* ≤ 0.0001). (**f**) Tissues from animals culled at 8, 21, 32, and 40 days were periodic acid–Schiff (PAS) and trichrome stained. Bar = 25 μm. In the mutant sections, the PAS staining clearly shows accumulation of extracellular matrix and developing fibrosis over the time course shown. Trichrome staining shows evidence of sclerosis. Representative images shown from electron microscopy studies indicate that the NPHS2 Cre PAR-1^Active+/−^ mouse is born with a normal phenotype. The ultrastructure of the filtration barrier is well maintained with clear podocyte foot processes. However, this ultrastructure is completely ablated by 26 days of age in the NPHS2 Cre PAR-1^Active+/−^ mice. (**g**) With podocyte foot process effacement (*) and thickening of the glomerular filtration barrier (#). Bar = 500 nm. A pathologist (MB) scored 48 images of murine glomeruli, 24 from each of Cre PAR-1^Active+/−^ mice and control mice. (**h**) The genotype of the mouse, be it control NPHS2 Cre PAR-1^Active+/−^, had a significant impact on the level of glomerulosclerosis (2-way analysis of variance, *P* ≤ 0.00001). (i) Collating the glomerulosclerosis scores also indicated that there was significantly more total sclerosis in the NPHS2 Cre PAR-1^Active+/−^ mice compared with wild type (Fisher’s exact test, *P* ≤ 0.00001). ACR, albumin to creatinine ratio. *P ≤ 0.05, **P ≤ 0.01, ***P ≤ 0.001, and ****P ≤ 0.0001. To optimize viewing of this image, please see the online version of this article at www.kidney-international.org.

A construct encoding a C-terminal phospho-null form of the PAR-1 receptor was cloned into the ROSA 26 locus of SV129 mice. This form of the PAR-1 receptor is constitutively active because it remains at the membrane once activated rather than being rapidly shuttled to the lysosome for degradation. We initially confirmed the specific location of PAR-1 in the podocyte by detecting the FLAG-tag protein specifically in these cells (Figure 4a).

The NPHS2 Cre PAR-1^Active+/−^ (developmental) mice expressed transgenic PAR-1 following cleavage by Cre recombinase (Supplementary Figure S3), were born at normal frequency, and were indistinguishable from their control littermates initially. Postnatally, the NPHS2 Cre PAR-1^Active+/ −^ mice became progressively more albuminuric. By 32 days of age, they were grossly albuminuric (Figure 4b). NPHS2 Cre PAR-1^Active+/−^ mice were also in renal failure as evidenced by their significantly higher levels of serum creatinine and urea compared with littermate controls (Figure 4c and d, respectively).

Furthermore, NPHS2 Cre PAR-1^Active+/−^ mice died between the ages of 39 and 45 days (Figure 4e). Littermate controls were healthy with normal lifespans.

Mice were culled at days 8, 21, 32, and 40. The day 8 NPHS2 Cre PAR-1^Active+/−^ mice had indistinguishable glomerular histology compared with controls (Figure 4f). By day 32 on both the periodic acid–Schiff and trichrome images, there was an accumulation of matrix within the glomerulus, and this matrix is largely composed of collagen. At day 32, the glomeruli of these animals were clearly fibrotic. By 40 days of age (where death from kidney failure in the mutants is imminent), there were clear signs of glomerulosclerosis. This progression of histologic features is reminiscent of human FSGS.

Electron microscopy studies demonstrated that at day 1 of age, the kidneys in the mutant mice appeared ultrastructurally normal. However, as early as day 26, the fine ultrastructure of the glomerular basement membrane was becoming deranged, with complete effacement of podocyte foot processes (Figure 4g).

It was found that only the PAR-1^Active+/−^ mice showed evidence of sclerosis, whereas the control animals showed no evidence of sclerosis (Figure 4h and i). There was a significant increase in sclerosis in the PAR-1^Active+/−^ mice when looked at by individual mouse and a significant increase in sclerosis when the mice were collated by genotype.

### Activation of podocyte PAR-1 in maturity also causes nephrotic syndrome

**Figure 5:**
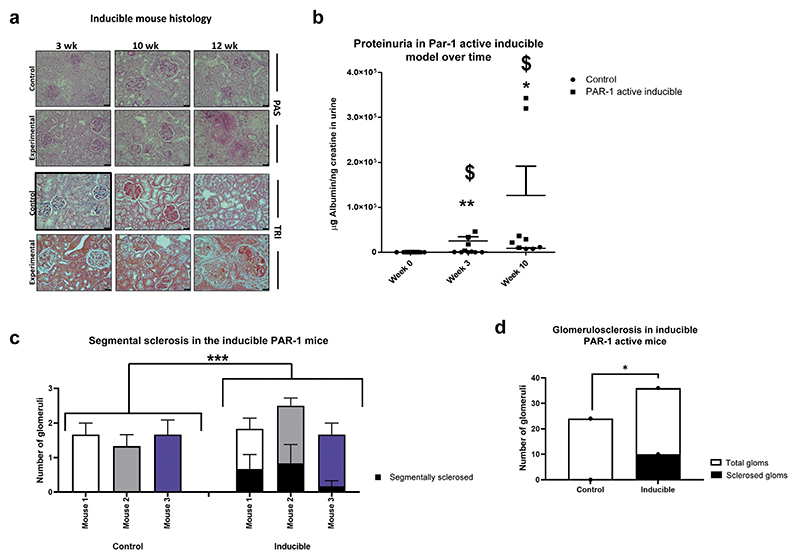
Activation of podocyte protease-activated receptor 1 (PAR-1) in maturity is also highly detrimental. The inducible mice, after induction with doxycycline (dox) treatment, develop a similar phenotype to the developmental model. (**a**) Albeit over a longer time course, the PAS staining indicates clear fibrosis by 12 weeks, with the trichrome staining showing sclerosis by the same time point. The PAR-1–active inducible animals demonstrate a significant increase in their albumin to creatinine ratio after treatment with doxycycline (2 mg/l 5% sucrose) for 3 weeks (^$^Kruskall-Wallis test, *P =* 0.0160). They are also significantly more proteinuric than their littermate controls who have been subjected to the same doxycycline treatment (**Mann-Whitney *U* test, *P =* 0.0043). The same significant interactions are seen at 10 weeks after the commencement of treatment; the PAR-1–active inducible animals maintain a significant increase in proteinuria compared with 0 weeks (^$^Kruskall-Wallis test, *P =* 0.0160), and they also remain significantly more proteinuric than their littermate controls (*Mann-Whitney *U* test, *P =* 0.0476). (**b**) The Pod rtTA Tet O Cre PAR-1 mice have been bred on a single SV129 background. A pathologist (MB) scored 6 mice, 3 control pod rtTA Tet O Cre -ve PAR-1^Active+/−^ and 3 pod rtTA Tet O Cre -ve PAR-1^Active+/−^ mice. Both groups received doxycycline treatment. (**c**) There was significantly more segmental sclerosis in the inducible mice than in the controls (2-way analysis of variance, *P* = 0.0297). (**d**) This significance held true when the data for the mice were pooled (Fisher’s exact test *P* = 0.0154). *P ≤ 0.05, **P ≤ 0.01, ***P ≤ 0.001, and ****P ≤ 0.0001. PAS, periodic acid–Schiff; TRI, trichrome. To optimize viewing of this image, please see the online version of this article at www.kidney-international.org.

A podocyte-specific inducible PAR-1^Active+/−^ mouse line was generated to investigate to what extent activation of podocyte PAR-1 in adult mice reproduces this phenotype. The mutants were induced at between 6 and 8 weeks of age with 2 mg/ml doxycycline. The inducible mutants demonstrated clear signs of NS within 3 weeks including significantly increased levels of albuminuria and glomerular mesangial expansion on periodic acid–Schiff stain (Figure 5a and b).

The progression of albuminuria and histologic changes were similar to the results seen in the developmental model, including an initial hypercellularity followed by mesangial expansion with fibrosis and sclerosis. Blind scoring by a pathologist (MB) revealed a significant increase in segmental sclerosis in the inducible mutants (Figure 5c and d).

### Signaling pathways in glomeruli of the developmental PAR-1 model mirror human plasma and biopsy findings

**Figure 6:**
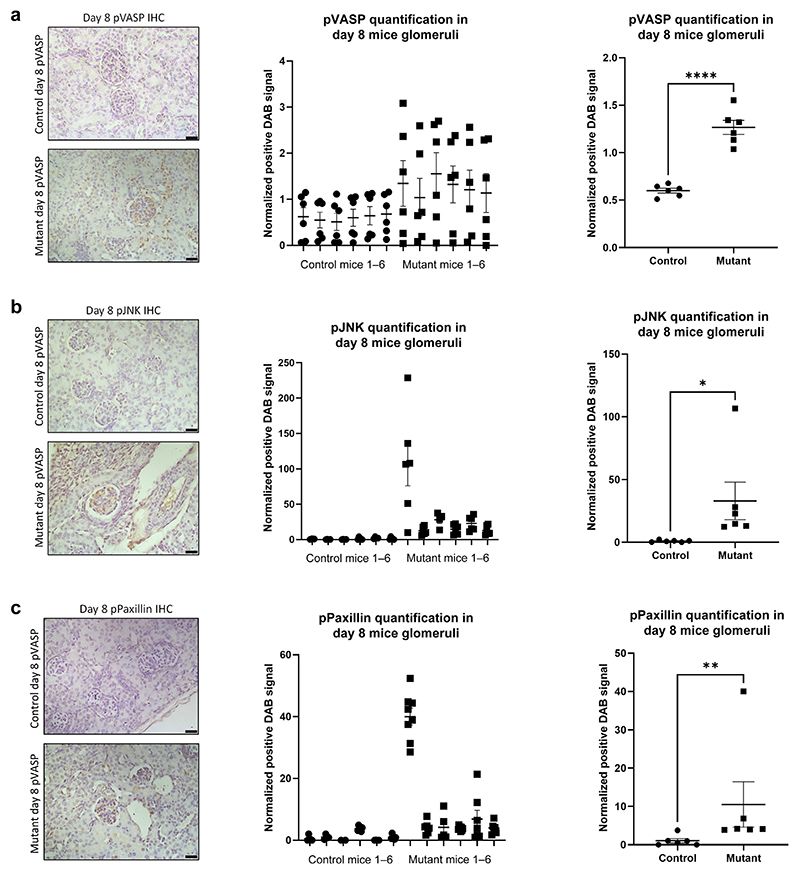
Protease-activated receptor 1 (PAR-1) signaling response in mouse model biopsies. Kidneys from NPHS2 Cre PAR-1^Active+/−^ mice and control mice were harvested from 8-day-old mice. These sections were stained for (a) Phospho–vasodilator-stimulated phosphoprotein (pVASP). There is significantly more pVASP in the glomeruli of Cre PAR-1^Active+/−^ mice relative to control mice (1-tailed unpaired *t* test, *P* ≤ 0.001). (b) Phospho–c-Jun N-terminal kinase (pJNK) was also measured and showed a significant increase in the glomeruli of Cre PAR-1^Active+/−^ mice (1-tailed unpaired *t* test, *P* ≤ 0.0291). (c) pPaxillin staining was also performed. Six glomeruli from 6 mice in each group were analyzed; the distribution of the 3,3′-diaminobenzidine (DAB) measurements for pPaxillin is shown in the middle panel. There is significantly more pPaxillin in the glomeruli of Cre PAR-1^Active+/−^ mice relative to control mice (1-tailed unpaired *t* test, *P* ≤ 0.0011). This is an *in vivo* validation of the *in vitro* data presented in Figure 1. *P ≤ 0.05, **P ≤ 0.01, ***P ≤ 0.001, and ****P ≤ 0.0001. The Pod Cre PAR-1 mice are on a single SV129 background. IHC, immunohistochemistry. To optimize viewing of this image, please see the online version of this article at www.kidney-international.org.

By IHC, there was a significant increase in VASP phosphorylation in the glomeruli of the NPHS2 Cre PAR-1^Active+/−^ mice at day 8 (Figure 6a). There were also significant increases in the staining of pJNK (Figure 6b), all seen in a podocyte distribution. pPaxillin signaling was also significantly increased in the glomeruli of the NPHS2 Cre PAR-1^Active+/−^ mice at day 8 (Figure 6c). We noticed that 1 of 6 PAR-1^Active+/−^ was a relative outlier for pJNK and pPaxillin, but the trend for all the rest was in the same direction. IHC was performed on kidney sections from 3 mice, with 6 to 8 glomeruli analyzed per mouse. This is the same signaling response as seen in the human podocytes *in vitro* in response to both PAR-1 agonist and relapse plasma.

### Detrimental effects of PAR-1 activation are partially mediated through TRPC6

**Figure 7:**
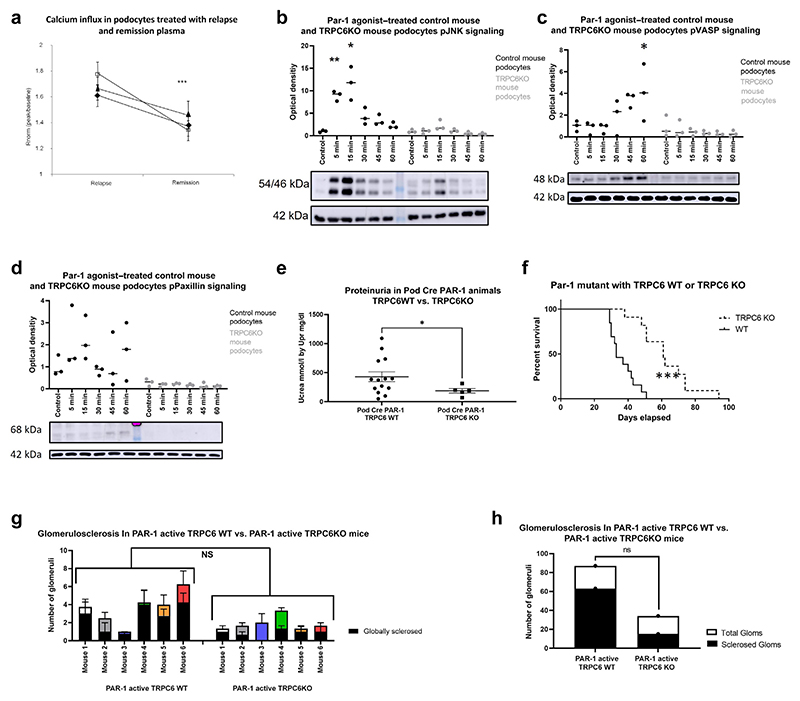
Detrimental effects of protease-activated receptor 1 (PAR-1) activation are mediated through TRPC6. Calcium influx was measured as a surrogate for transient receptor potential cation channel subfamily c member 6 (TRPC6) activity. R_norm_ shows the level of calcium influx after 15 μM flufenamic acid treatment. (**a**) Podocytes treated with relapse plasma saw a significant increase in calcium influx compared with podocytes treated with remission plasma. This suggests the presence of a factor in the relapse plasma that can potentiate the activity of the TRPC6 calcium channel. (**b**–**d**) Treatment of wild-type (WT) mouse podocytes with PAR-1 agonist replicated the responses seen in human WT podocytes (Bonferroni’s multiple comparison test). TRPC6 knockout (KO) podocytes were treated with PAR-1 agonist for the indicated time points. These podocytes did not show the signature response of (**b**) phospho–c-Jun N-terminal kinase (pJNK), (**c**) phospho–vasodilator-stimulated phosphoprotein (pVASP), and (**d**) pPaxillin. (**e**) TRPC6 was knocked out of the Pod Cre PAR-1–active mice (see the Methodssection for model generation); these mice were significantly less proteinuric at 42 days (1-way Mann-Whitney test, *P =* 0.0402). (**f**) Pod Cre PAR-1^Active+/−^ TRPC6 KO mice lived significantly longer (TRPC6 WT n = 13, median survival 33 days vs. 7.2 days; TRPC6^−/−^ n = 11, median survival 61 days vs. 14.7 days Mantel-Cox test, *P* ≤ 0.0001). The Pod Cre PAR-1 mice were crossed with whole-body TRPC6 KO mice on a C57/Bl6. A pathologist (MB) scored 6 Pod Cre PAR-1^Active+/−^ mice on the mixed SV129/C57Bl/6 background versus their Pod Cre PAR-1^Active+/−^ TRPC6 KO counterparts and found no significant difference in glomerulosclerosis. *P ≤ 0.05, **P ≤ 0.01, ***P ≤ 0.001, and ****P ≤ 0.0001. These data can be seen by (**g**) individual mouse or (**h**) collated.

Flufenamic acid is a specific TRPC6 agonist that stimulates calcium influx in podocytes.^23^ This calcium influx was potentiated by treatment with relapse plasma from nephrotic patients compared with remission plasma (Figure 7a).

To test if TRPC6 mediates podocyte signaling pathways downstream of PAR-1, a TRPC6-null mouse podocyte cell line was treated with PAR-1 agonist peptide.^24^ Significant phosphorylation of JNK and VASP was seen in WT mouse podocytes with no significant phosphorylation in TRPC6 KO podocytes (Figure 7b and c, respectively). Similarly, for paxillin, there seemed to be evidence of stimulation of the WT podocytes after PAR-1 agonist treatment with no evidence of stimulation of paxillin in the TRPC6 KO mice (Figure 7d). It is clear that the TRPC6 KO podocytes demonstrate a blunted promotility response to PAR-1 agonist treatment.

*In vivo*, we crossed the Pod Cre PAR-1^Active+/−^ mouse with a TRPC6^−/−^ mouse to generate the developmental PAR-1^Active^ mouse on a TRPC6-null background. The whole-body KO of TRPC6 can exacerbate glomerular disease but is not sufficient to cause disease.^25^ The TRPC6 KO mice crossed with PAR-1^Active^ had significantly reduced proteinuria compared with PAR-1^Active^ mice (Figure 7e). TRPC6 KO in the Pod Cre PAR-1^Active+/−^ significantly improved lifespan (Figure 7f). These animals are on a mixed background including the C57 Bl6 strain, which are known to be resistant to proteinuria. Indeed, the control mice on this mixed background are less proteinuric than their counterparts on a singleSV129 background. Blind scoring by a pathologist (MB) revealed no significant change in glomerulosclerosis between the WT and TRPC6KO variants of the PAR-1–active mice (Figure 7g and h).

### TRPC6KO Pod Cre PAR-1^Active+/−^ mice show significantly reduced glomerular pVASP and retain good filtration barrier ultrastructure

**Figure 8:**
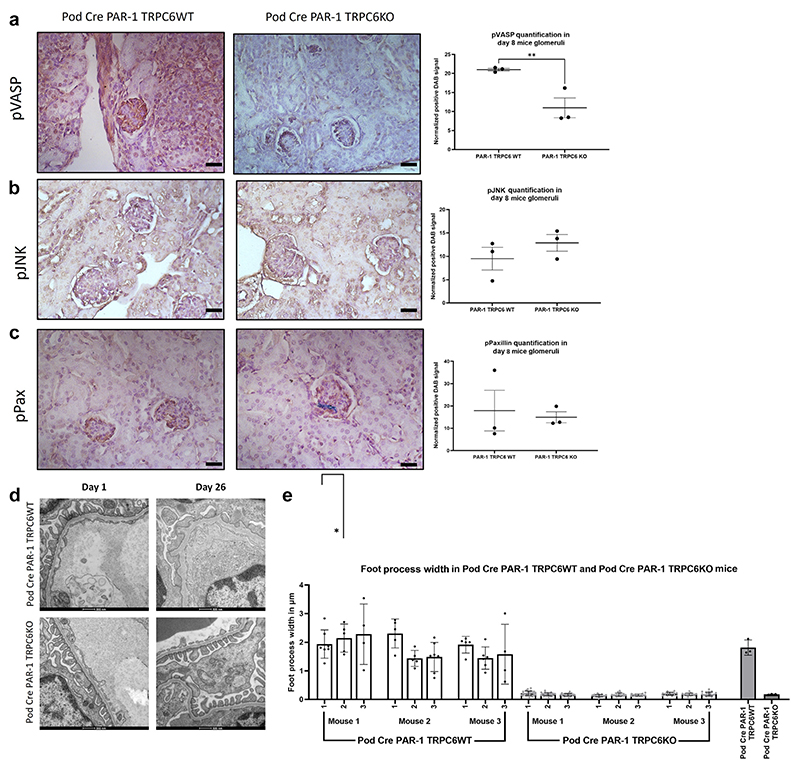
Transient receptor potential cation channel subfamily c member 6 (TRPC6) knockout (KO) significantly reduces glomerular phospho–vasodilator-stimulated phosphoprotein (pVASP) and protects filtration barrier ultrastructure. Glomerular signaling in the Pod Cre protease-activated receptor 1 (PAR-1)^Active+/−^ TRPC6 wild-type (WT) and Pod Cre PAR-1^Active+/−^ TRPC6 KO was interrogated using immunohistochemistry (IHC) targeted against (**a**) phospho–vasodilator-stimulated phosphoprotein (pVASP), (**b**) phospho–c-Jun N-terminal kinase (pJNK), and (**c**) pPaxillin. Only pVASP demonstrated a significant difference between the 2 models. Glomerular pVASP was significantly reduced in the TRPC6 KO variant compared with the TRPC6 WT (*P =* 0.0093, 1-tailed unpaired *t* test). (**d**) Representative images shown from electron microscopy (EM) studies that revealed that the structure of the filtration barrier was deranged by 26 days in the Pod Cre PAR-1^Active+/−^ TRPC6 WT mice. Additional representative images can be seen in Supplementary Figure S4. (e) TRPC6 KO in the Pod Cre PAR-1^Active+/−^ mice leads to retention of the neat, highly regulated structure of the glomerular filtration barrier. Indeed, analysis of the EM demonstrated a significant decrease in foot process width between TRPC WT and TRPC6 KO variants of the Pod Cre PAR-1^Active+/−^ model (1-tailed Mann-Whitney test, *P =* 0.0500). *P ≤ 0.05, **P ≤ 0.01, ***P ≤ 0.001, and ****P ≤ 0.0001. DAB, 3,3′-diaminobenzidine. To optimize viewing of this image, please see the online version of this article at www.kidney-international.org.

There was a significant reduction in glomerular pVASP signaling in the mice at 8 days in the Pod Cre PAR-1^Active+/−^ TRPC6KO mice versus the Pod Cre PAR-1^Active+/−^ TRPC6 WT mice (Figure 8a). There was no significant difference between the 2 models in respect of pJNK and pPaxillin signaling pathways (Figure 8b and c).

Despite the change of background, the Pod Cre PAR-1^Active+/−^ mice had a normal filtration barrier at day 1, with a complete ablation of the barrier by day 26. Pod Cre PAR-1^Active+/−^ TRPC6 KO mice retained tight regulation of the structure of the filtration barrier (Figure 8d). Measuring the width of the podocyte foot processes in these mice revealed that knocking out TRPC6 in the mice caused a significant reduction, indicative of significantly less foot process effacement (Figure 8e).

## Discussion

The PAR-1 receptor is enriched in podocytes of the kidney cortex. The treatment of human podocytes *in vitro* with PAR-1 agonist and active human nephrotic plasma leads to consistent activation of the same pathways (VASP, JNK, and p38 MAPK). Remission plasma elicited a much smaller response. This suggests that there could be an effector molecule enriched in relapse plasma that stimulates a signature signaling response via the PAR-1 receptor. Indeed, work published by colleagues has established that inhibition of PAR-1 using vorapaxar can block the signaling response of the podocyte to nephrotic plasma.^26^ This signaling response is partially dependent on TRPC6. The podocyte-specific VASP and JNK signaling response *in vitro* was also observed in human nephrotic patient biopsies. Moreover, the JNK and VASP signaling was significantly increased in biopsies of patients with clinically likely circulating factor disease. Patient 643 has Frasier syndrome, a genetic form of FSGS. Though histologically similar to FSGS, it is not caused by a circulating factor; there was no evidence of VASP or JNK signaling in this patient.

The developmental mouse model replicated the signature signaling response. Additionally, they developed a histology that progressed over time and was reminiscent of human FSGS. The NPHS2 Cre PAR-1^Active+/−^ mice died of renal failure around 40 days of age. We were able to significantly reduce proteinuria and increase lifespan of PAR-1^Active+/−^ mice by 50%, by TRPC6 KO. This identifies TRPC6 as an important part of the PAR-1 downstream signaling pathway. However, blind scoring by a pathologist (MB) of periodic acid–Schiff and trichrome images revealed no significant differences in the levels of glomerulosclerosis.

These data suggest that the signaling from the PAR-1 receptor to JNK and Paxillin is via TRPC6. KO of TRPC6 blocks the downstream phosphorylation of JNK and Paxillin when PAR-1 is activated. The TRPC6 KO PAR-1 mice live longer than the PAR-1 TRPC6 WT mice but still die prematurely of renal failure. This suggests that phosphorylation of both JNK and VASP downstream of PAR-1 activation is deleterious.

A role for TRPC6 in PAR-1–mediated endothelial contraction has also been reported. In pulmonary endothelial cells, TRPC6 mediates RhoA activity and endothelial cell contraction downstream of activation of protein kinase Cα in response to thrombin.^25,27^

Studies have also implicated PAR-1 activation to have a role in epithelial permeability, for example, in the intestine.^28^ Its role in regulating glomerular permeability via the podocyte would align with these known functions and has never previously been described.

We have identified a receptor that we believe a circulating factor could work through. We show evidence that the same signaling pathways identified *in vitro* in response to PAR-1 agonist and to patient plasma are present and enriched in human nephrotic patient biopsies. Correspondingly, the signaling signature was present in the NPHS2 Cre PAR-1^Active+/−^ mice. Together these findings demonstrate that overactivation of the PAR-1 receptor can induce an NS that is histologically similar to human FSGS and have identified a common signature signaling response that could be used to stratify patients and isolate those with circulating factor disease.

Other groups have shown that mice treated with PAR-1 agonists developed a more severe phenotype in a glomerulonephritis model.^29^ The PAR-1 antagonist Q94 is known to protect against nephropathy in adriamycin-treated mice.^10^ On the other hand, PAR-1 KO mice do not develop streptozotocin-induced glomerular damage.^30^ What is not tested in the current literature is the result of PAR-1 receptor overactivation. Despite Sharma *et al*.^30^ postulating that it is PAR-3 and PAR-4 that are important in human podocytes and disease, we show convincing evidence that the PAR-1 receptor can stimulate pathologic signaling and changes in human podocytes.^31^

This work does not identify or prove that the causative circulating factor(s) is a protease but provides insight into the mechanism of the circulating factor disease, leading to the following hypothetical pathogenesis. During active disease (e.g., inflammation, endothelial damage and activation, etc.) there is a spike in activated proteases in the circulation from a number of potential biological cascades. These serine proteases cleave and activate the PAR-1 receptor inducing downstream signaling in the podocytes that generate a promigratory phenotype. This constant activation of PAR-1, as replicated by our model, has a detrimental effect on podocyte dynamics and the function of the glomerular filtration barrier. Ultimately, the barrier fails and the hallmark features of FSGS are observed: increasing fibrosis and sclerosis of the glomeruli, massive proteinuria along with eventual renal failure.

Recently, we hypothesized that the Th17 subset of T cells, that are associated with several inflammatory diseases, could be secreting proteases as putative circulating factors. The active phase of minimal change disease has been linked to an increase in interleukin-17A producing Th17 cells, and an increase in Th17 cells and their markers has also been reported during active disease.^32^ We have shown that human Th17 cell supernatant induces the same promotility signaling pathways in cultured podocytes that can be blocked by a PAR-1 inhibitor, namely phosphorylated JNK, paxillin, and p38MAP kinase.^6^ In this paper, we present a putative *in vivo* model of circulating factor disease, which will be invaluable in further elucidating disease pathogenesis.

We have pinpointed the importance of PAR-1 receptor signaling in the pathogenesis of INS. In the process we have proposed a model of circulating factor disease that will be invaluable in developing novel treatment strategies.

## Supplementary Material

Supplementary Material

## Translational Statement

There is compelling evidence implicating the role of a circulating factor in nephrotic syndrome, and we have previously shown that this factor signals via protease-activated receptor 1 (PAR-1). This work further extends our previous data showing that the signaling responses to PAR-1 agonists were conserved between 3 systems: podocytes *in vitro*, the mouse model expressing a transgenic overactive form of the PAR-1 receptor, and human circulating factor disease, demonstrating the significance. Looking downstream, TRPC6 (transient receptor potential cation channel subfamily c member 6) knockout podocytes demonstrated altered PAR-1 agonist signaling responses. Knockout of TRPC6 in the PAR-1–active mice significantly reduced their proteinuria and extended their lifespan. This work suggests that PAR-1 could be a key target in the treatment of nephrotic syndrome.
