# Supplementary material for: Podocyte protease activated receptor 1 stimulation in mice produces focal segmental glomerulosclerosis mirroring human disease signaling events
Source: Kidney Int. Author manuscript; Available in PMC 2024 Aug 7. (PMC7616342; doi:10.1016/j.kint.2023.02.031)
Supplement: Supplementary Material [file EMS197722-supplement-Supplementary_Material.pdf]

SUPPLEMENTARY MATERIAL

Supplementary File (PDF)

Supplementary Figure S1. Glomerular endothelial cell (*GEnC*) protease-activated receptor 1 (*PAR-1*) signaling response. GEnC were treated with 15 μM PAR-1 agonist. There was no phosphorylation of Paxillin, vasodilator-stimulated phosphoprotein (VASP), or c-Jun N-terminal kinase (JNK) in response to PAR-1 agonist. Phosphorylation of p38 mitogen-activated protein kinase (MAPK) was observed in response to PAR-1 agonist treatment. This shows that the GEnC can respond to PAR-1 agonist and that the p38 MAPK signaling response is not podocyte specific, suggesting that the other studied pathways are more relevant for the podocyte. Positive control is wild-type human podocytes treated with 15 μM PAR-1 agonist for 15 minutes.

Supplementary Figure S2. Patient biopsy clinical information.

Supplementary Figure S3. Protease-activated receptor 1 (PAR-1) active construct. The PAR-1 active construct is downstream of a CAGG promoter inserted at the Rosa 26 locus within the mouse genome. Upstream of the sequence encoding the transgenic PAR-1 receptor is a cassette containing a stop codon and the neomycin selection marker. The stop codon and neomycin selection marker is flanked by loxP sites. Cre recombinases cleave the DNA at loxP sites and stiches the ends together. When Cre recombinase acts upon the Rosa 26 locus, the sequence containing the stop codon and neomycin selection marker is removed and the PAR-1–active transgene is expressed. In the developmental model, Cre recombinase is expressed under the control of a podocin promoter, whereas in the inducible model, Cre recombinase is expressed after treatment with doxycycline.

Supplementary Figure S4. Additional representative images for Pod Cre protease-activated receptor 1 (PAR-1)$^{Active\ +/-}$ transient receptor potential cation channel subfamily c member 6 (TRPC6) wild type (WT) and Pod Cre PAR-1$^{Active+/-}$ TRPC6 knockout (KO) electron microscopy. The 6 images shown in the figure are from 6 different mice (3 Pod Cre PAR-1$^{Active+/-}$ TRPC6 WT and 3 Pod Cre PAR-1$^{Active+/-}$ TRPC6 KO). They are representative of the pathology seen in these animals.
